# Metabolic Fingerprint Analysis of Cytochrome *b*_5_-producing *E. coli* N4830-1 Using FT-IR Spectroscopy

**DOI:** 10.3389/fmicb.2022.874247

**Published:** 2022-06-22

**Authors:** Thanyaporn Tengsuttiwat, Naheed Nazly Kaderbhai, Joe Gallagher, Royston Goodacre, Howbeer Muhamadali

**Affiliations:** ^1^Centre for Metabolomics Research, Department of Biochemistry and Systems Biology, Institute of Systems, Molecular and Integrative Biology, University of Liverpool, Liverpool, United Kingdom; ^2^Institute of Biological, Environmental and Rural Sciences, Gogerddan Campus, Aberystwyth University, Aberystwyth, United Kingdom

**Keywords:** FT-IR spectroscopy, metabolic fingerprint, recombinant protein production, cytochrome *b*_5_, chemometrics

## Abstract

Optimization of recombinant protein expression in bacteria is an important task in order to increase protein yield while maintaining the structural fidelity of the product. In this study, we employ Fourier transform infrared (FT-IR) spectroscopy as a high throughput metabolic fingerprinting approach to optimize and monitor cytochrome *b*_5_ (CYT *b*_5_) production in *Escherichia coli* N4830-1, as the heterologous host. Cyt b_5_ was introduced as a plasmid with between 0 and 6 copies under a strong promoter. The FT-IR spectroscopy results combined with multivariate chemometric analysis illustrated discriminations among culture conditions as well as revealing features that correlated to the different *cytb*_5_ gene copy numbers. The second derivative of the FT-IR spectral data allowed for the quantitative detection of Cyt b_5_ directly inside the intact cells without the need for extraction, and highlighted changes in protein secondary structure that was directly correlated to the *cytb*_5_ gene copy number and protein content, and was in complete agreement with quantitative findings of standard traditional techniques such as SDS–PAGE and western blot analysis.

## Introduction

Recombinant protein production has played an important role for decades in a wide range of industries, i.e., pharmaceuticals, diet, and cosmetics (Kirk et al., 2002; Singh et al., 2016). Various hosts have been used for this purpose, ranging from prokaryotes such as *E. coli* and *Streptomyces* spp. to eukaryotic cells, namely, the yeasts *Saccharomyces cerevisiae* and *Komagataella phaffii* known as *Pichia pastoris* (Vieira Gomes et al., 2018). Despite its known limitations, *E. coli* is still a popular host due to the fact that it is easy to handle, genetically tractable requires simple media, grows rapidly, and has been developed over many years to be a versatile host that can be used in a wide range of applications (Leis et al., 2013). However, the development of bacterial hosts for the expression of recombinant proteins more efficiently is still investigated by various approaches such as the optimization of culturing conditions and genetic engineering methods, which have been discussed in detail in an excellent review by Tripathi and Shrivastava (2019). A good example of the latter approach is the modification of mRNA stability and translational efficiency leading to the development of a BL21 derivative commercial vector known as Star™ (Lopez et al., 1999; Makino et al., 2011).

In the past two decades, metabolomics has also been applied as a useful approach for the optimization of recombinant expression systems, as the study of the metabolome, the building blocks of various biomolecules including proteins, may provide a clearer picture on what biochemical changes occur in the system compared with a genomic approach (Fiehn, 2002). Metabolomics can be described as the study of low molecular weight compounds that are usually primary metabolites involved in necessary bioprocesses that are essential for maintenance and growth of cells (Dunn and Ellis, 2005; Hollywood et al., 2006; Mashego et al., 2007). To synthesize secondary metabolites, energy such as ATP, precursors, cofactors, and other relevant building blocks, which are biosynthesized from simple primary metabolites, are required (Bailey, 1991). Therefore, the link between secondary metabolites, recombinant proteins, and low molecular weight compounds is worth studying as the availability of these metabolites may directly influence the biosynthetic rate of the desired recombinant protein (Stephanopoulos and Vallino, 1991). Furthermore, since both quality and quantity of the recombinant protein may be affected by the presence and maintenance of a foreign DNA, understanding the metabolic burdens of carrying such DNA material using metabolomics approach is also useful for the improvement of the recombinant protein production process (Muhamadali et al., 2015, 2016).

While a range of analytical techniques have been applied in metabolomics, mass spectrometry (MS) especially coupled with chromatographic methods is the most frequently used as it provides high sensitivity, selectivity, accuracy, and resolution (Kaderbhai et al., 2003; Dunn and Ellis, 2005; Dunn et al., 2011, 2013). However, MS-based metabolomics approaches are costly and require laborious sample preparation and complicated data interpretation which are time-consuming. Unlike MS, infrared (IR) spectroscopy is a versatile, non-destructive, and rapid method that can be used as a fingerprinting technique and requires minimum sample preparation (Dunn and Ellis, 2005). A good example is its application for classification and characterization of bacteria accurately and reproducibly down to sub-species level (Naumann et al., 1991). Since it is a high-throughput screening method, FT-IR spectroscopy has been applied to study the overall phenotypic changes of bacteria in different culture conditions (Dunn and Ellis, 2005; Muhamadali et al., 2015, 2016). FT-IR has also been used to study the secondary structure of proteins such as globular proteins structures in various concentration of ionic liquids to understand the protein stabilization (Arunkumar et al., 2019) and for quantitative study of protein structure in solution (Arrondo et al., 1993). Thus, in this work, FT-IR spectroscopy was applied to appraise the reproducibility of the production process and to optimize the protein production by varying the induction time of the λ*P*_L_ promoter. The FT-IR fingerprint results combined with chemometrics analysis, such as PCA and PC–DFA, was also employed to investigate the metabolic changes that occur in the expression system.

## Materials and Methods

### Bacterial Strains and Culture Conditions

In total, seven strains of *E. coli* N4830-1 (F^−^*suo thi-1 thr-1 leuB6 lacY1 fhuA21 supE44 rfbD1 mcrA1 his ilv galK8* Δ(*hemF-exp*) Δ(*bio uvrB*) [λ Δ*Bam* N^+^*c*I857 Δ(CroattR)]) harboring pEX-CYT plasmid were kindly provided by Dr. Naheed Kaderbhai. The strains carry different copy numbers of *cytb*_5_ gene ranging from 0 to 6 which are labeled as N0 to N6 (*E. coli* pλ-0*cyt* to *E. coli* pλ-6*cyt*). The vector pEX-CYT also contains an ampicillin-resistant gene as a selectable marker (Supplementary Figure S1). All the strains were streaked onto LB agar (Formedium, UK) prior to growing overnight at 37°C on 75 μg/mL ampicillin-supplemented LB agar for selection of the desired colonies.

### Bacterial Growth Profile

A single colony was inoculated into 5 mL of LB broth supplemented with 75 μg/mL ampicillin in a 50 mL sterile conical tube and incubated overnight at 37°C with 200 rpm shaking. The inoculum was then diluted with the medium to a final OD_600nm_ of 0.1 prior to transferring as 200 μL aliquots to a sterile 100 well-plate (10 replicates). Bacterial growth was then monitored with a Bioscreen C analyzer (Oy Growth Curves Ab Ltd, Finland) using the following settings: 5 min preheating, continuous medium shaking, OD_600nm_ measurement with 10 min intervals, and incubation at 37°C for 24 h.

### Cyt b_5_ Production

The cells were cultured for the Cyt b_5_ production following the procedure described by Kaderbhai et al. (1992). Briefly, 1 mL overnight culture was inoculated into a 250 mL Erlenmeyer flask containing 25 mL of LB medium plus 75 μg/mL ampicillin. The cells were incubated at 150 rpm and 30°C until reaching the mid-log phase (~2.5 h), then the incubation temperature was immediately increased to 38.5°C to activate the heat-sensitive promoter, λ*P*_L_. The culture was induced and incubated for 8 h to allow for the protein production, while a control set was kept uninduced and continuously incubated at 30°C. The final biomass for each sample was determined by measuring the OD_600nm_ afterward. To optimize the production condition, the bacteria were also cultured as 3 mL aliquots in 15 mL tubes for various lengths of time ranging from 8 to 14 h.

### FT-IR Fingerprint Analysis

#### Sample Preparation

Bacterial biomass was harvested by centrifugation of 1 mL samples at 5,000 *g* for 5 min at room temperature, followed by a washing step with 0.9% NaCl and normalization to the OD_600nm_ of 15 (Muhamadali et al., 2016). A 20 μL aliquot of each sample was spotted onto an FT-IR silicon plate that had been cleaned as described previously (Muhamadali et al., 2016); the locations of these samples were randomized. The plate was then dried in a 55°C oven for about 30 min. Samples were analyzed using an Invenio infrared spectrophotometer equipped with an HTS-XT high throughput plate reader (Bruker Optics Ltd., Coventry, UK) in transmission mode, with a spectral resolution of 4 cm^−1^ and within the 4,000–400 cm^−1^ range. Each sample class contained 3 biological replicates, and from each of the dried spots 4 machine replicates were collected to capture any variation in the bacterial population.

#### Data Analysis

The FT-IR data were collected with the OPUS spectroscopy software supplied by the manufacturers (Bruker UK Ltd., UK) and further analyzed using MATLAB^®^ (version r2019b, The MathWorks Inc., UK). All the spectra were primarily preprocessed as described by Muhamadali (Muhamadali et al., 2015). In brief, the extended multiplicative signal correction (EMSC) method (Martens et al., 2003) was applied to scale the spectra and remove any variations according to the sample size. Subsequently, the CO_2_ vibrations were removed from all spectra by replacing these regions with linear trends, prior to being analyzed using principal component analysis (PCA) which is an unsupervised method (Wold et al., 1987). These PCA data were further analyzed using discriminant function analysis (DFA) (Gromski et al., 2015). In this work, PCA combined with DFA (PC–DFA) was employed as a semi-supervised approach as the algorithm was only provided with information on the group of each bacterial strain excluding any relation or order from one to another group (i.e., the algorithm was not provided with the *cytb*_5_ gene copy number).

### Detection and Identification of CYT b_5_

The normalized biomass (~1 mL) of each sample were collected by centrifugation (5,000 *g*) for 10 min at room temperature and the supernatant was then kept separately to examine any protein leakage. The pellets were then prepared for analysis using sodium dodecyl sulfate-polyacrylamide gel electrophoresis (SDS–PAGE) following the protocol described previously (Kaderbhai et al., 1992). Subsequently, 15 μL of each sample was loaded into sample wells of a 15% polyacrylamide gel and 5 μL of PageRuler plus Prestained Protein Ladder (Thermo Fisher Scientific Inc., USA) was used as a protein marker. Electrophoresis was performed at 180 V for 50 min. The protein bands were subsequently subjected to western blot analysis, and transferred to a polyvinylidene fluoride (PVDF) membrane by applying a constant current at 400 mA for 1 h. The antibodies for the Cyt b_5_ target protein and the loading control, β-actin were purchased from Invitrogen (Thermo Fisher Scientific Inc., USA) and the detection reagents from Pierce ECL Western Blotting Substrate (Thermo Fisher Scientific Inc., USA). The target protein bands were detected chemiluminescently using ImageQuant LS 4000 (GE Healthcare UK Ltd, UK).

### Relative Quantification of Cyt b_5_

#### SDS–PAGE: Quantification Using a Total Protein Normalization

The total protein bands in the samples has been used for normalization purposes to allow for more accurate quantification of the target protein band (Gilda and Gomes, 2013). SDS–PAGE was used to monitor the relative quantity of the Cyt b_5_ content by loading samples in a random order and using the total protein bands for normalization. The densitometric data were calculated using ImageJ (Rueden et al., 2017).

#### Colorimetric Method: UV-Visible Spectroscopy

The Cyt b_5_ content in bacterial cells was monitored *via* spectrophotometry and focused on a range of 400–450 nm where a Soret absorption is detected (Kaderbhai et al., 1992). In addition to the previous study, external hemin was added to increase the reliability of the measurement as there are extra *cytb*_5_ genes in the system. Therefore, the amount of hemin naturally produced by the *E. coli* may not be adequate to incorporate into the protein. Since the red or pink color is from the incorporation of heme into the protein providing a Soret absorption in UV-vis region (Beck von Bodman et al., 1986; Pyrih et al., 2014). A stock solution of 1 mM hemin was prepared in 0.1 M NaOH (65.2 mg/mL), followed by a centrifugation step to remove any residues, and measuring the absorbance at 385 nm and calculating the concentration with an extinction coefficient of 58.4 mM^−1^. Hemin solution was then added into an aliquot (200 μL) of cell lysates to a final concentration of 0, 5, 10, 20, 40, 60, 80, and 100 μM. The UV-vis spectra were measured with the lysing solution (TE buffer with 0.1 mg/mL lysozyme) supplemented with hemin as a reference at each concentration.

## Results

### Bacterial Growth Profile

**Figure 1:**
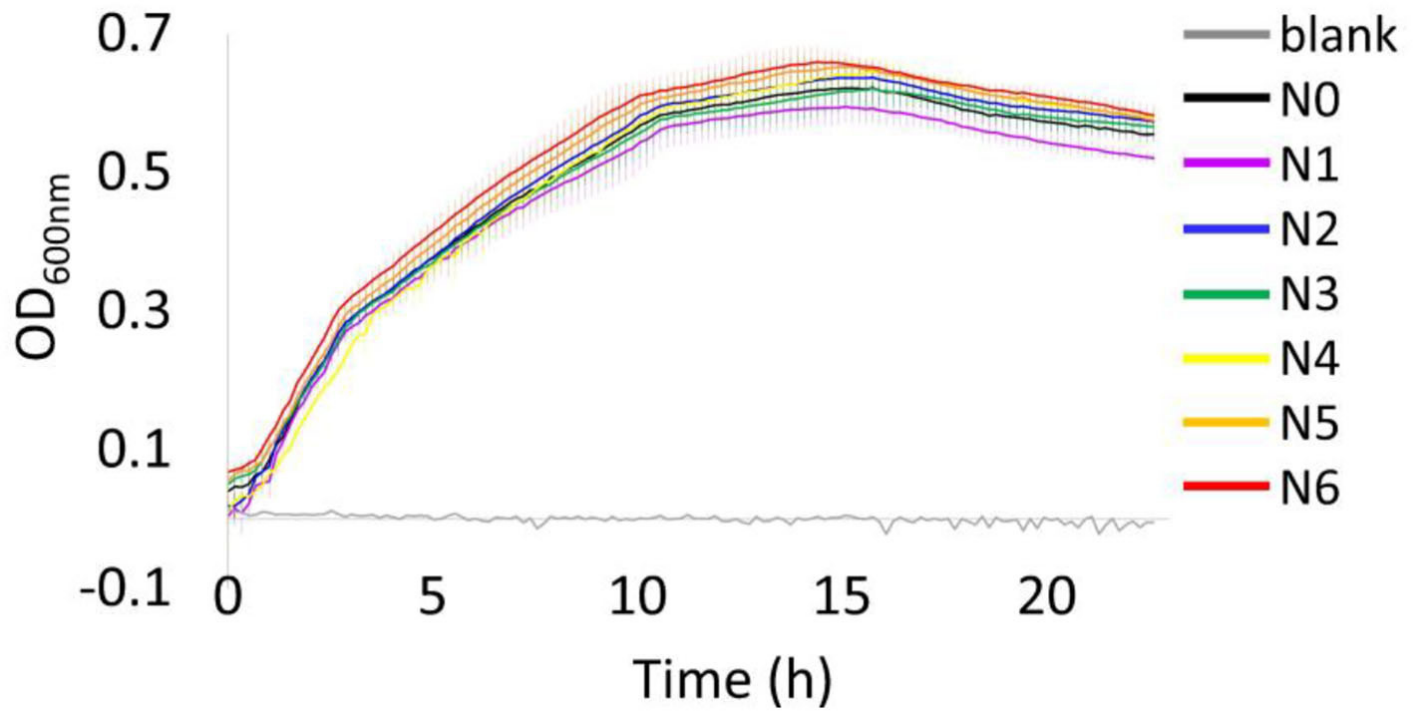
Growth profiles of *E. coli* N4830-1 strain N0–N6 cultured at 37°C in LB medium (75 μg/mL ampicillin) with a blank, LB medium (75 μg/mL ampicillin) using a Bioscreen C analyzer. Values are means and error bars showing the SD (from 10 biological replicates).

Overall, the growth behavior of all bacterial strains under 37°C were similar throughout the incubation period, with all strains reaching the stationary phase at around 10 h (Figure 1). At the end of the incubation period (24 h), a decrease in OD_600nm_ has been observed that may indicate the start of death phase. The bacterial strain N1 showed the lowest OD_600nm_ value when they reached the stationary phase, while strain N6 displayed as the highest OD_600nm_. However, these are insignificantly different as illustrated by the error bars showing the SD of each strain (calculated from 10 biological replicates, *n* = 10).

### Cyt b_5_ Production

All the bacterial strains reached an average final OD_600nm_ of 3.5. The pH of the media for all samples were specifically monitored to ensure that any changes detected in the FT-IR metabolic fingerprint of the cells were because of Cyt b_5_ production and not a result of any environmental changes. The pH values displayed similar changes for all the examined strains and changed from ~6.8 to around 8.2 (average values) after induction (Supplementary Table S1). This alkalinization of the medium is expected due to the cells being cultured in a rich medium where amino acids are catabolized (deamination process), to be used as a carbon source followed by the release of the generated ammonium into the surrounding medium. However, the harvested bacterial cells were visible as pale pink color in some strains (N3–N6) as shown in Supplementary Figure S2.

### FT-IR Fingerprint Analysis and the Optimization of Inducing Time for Cyt b_5_ Production

The FT-IR spectra of induced *E. coli* N4830-1 strain after pre-processing displayed the commonly known vibrational regions of bacterial cells (Table 1), i.e., the region between 1,200 and 900 cm^−1^ absorption bands of nucleic acids (${\text{PO}}_{2}^{-}$) and bacterial cell wall component such as polysaccharides (C-O-C) (Zarnowiec et al., 2015). The 1,700–1,500 cm^−1^ region is particularly important as it is usually the most intense region and provides information on the amide I (C=O stretching) and II (C–N stretching, N–H bending) vibrational bands that are the vital chemical characteristic of peptide bonds in proteins (Zarnowiec et al., 2015). The second derivative of the FT-IR spectral data illustrated the underlying peaks as shown in Supplementary Figure S3 that can be assigned to the secondary structure of proteins, i.e., the 1,631 cm^−1^ region belongs to the β-sheet structure of amide I and 1,658 cm^−1^ represents the α-helix structure (Holloway and Mantsch, 1989; Kong and Yu, 2007). The vibration in amide II region at about 1,567 cm^−1^ also relates to the β-sheet structure of the protein (Brian et al., 2014). Amide bands of proteins were also observed in the uninduced set; however, its second derivative spectra (Supplementary Figure S3A) showed similar intensities among all strains, while there was variation in the induced set (Supplementary Figure S3B). This band intensity variation among the 7 strains in Cyt b_5_ production condition (38.5°C), especially at 1,658 cm^−1^ (Supplementary Figure S3C), indicates a difference in the level of the protein content and their structure under the examined condition.

**Table 1:** Vibrational regions and possible band assignment.

**Wavenumber (cm^**−1**^)**	**Possible band assignment**	**Vibration**
3,000–2,800	Lipids	C-H stretching
1,700–1,600 1,684 1,658 1,639 1,631	Amide I β-sheet α-helix β-sheet β-sheet	C=O and C-N stretching
1,600–1,500 1,567	Amide II β-sheet	N-H bending and C-N stretching
1,500–1,200	Amide III, fatty acids, and phosphate carrying compounds	N-H bending, C-N stretching, and C-H bending
1,200–900	Nucleic acids and polysaccharides	P=O, C=O, and C-O-C stretching

**Figure 2:**
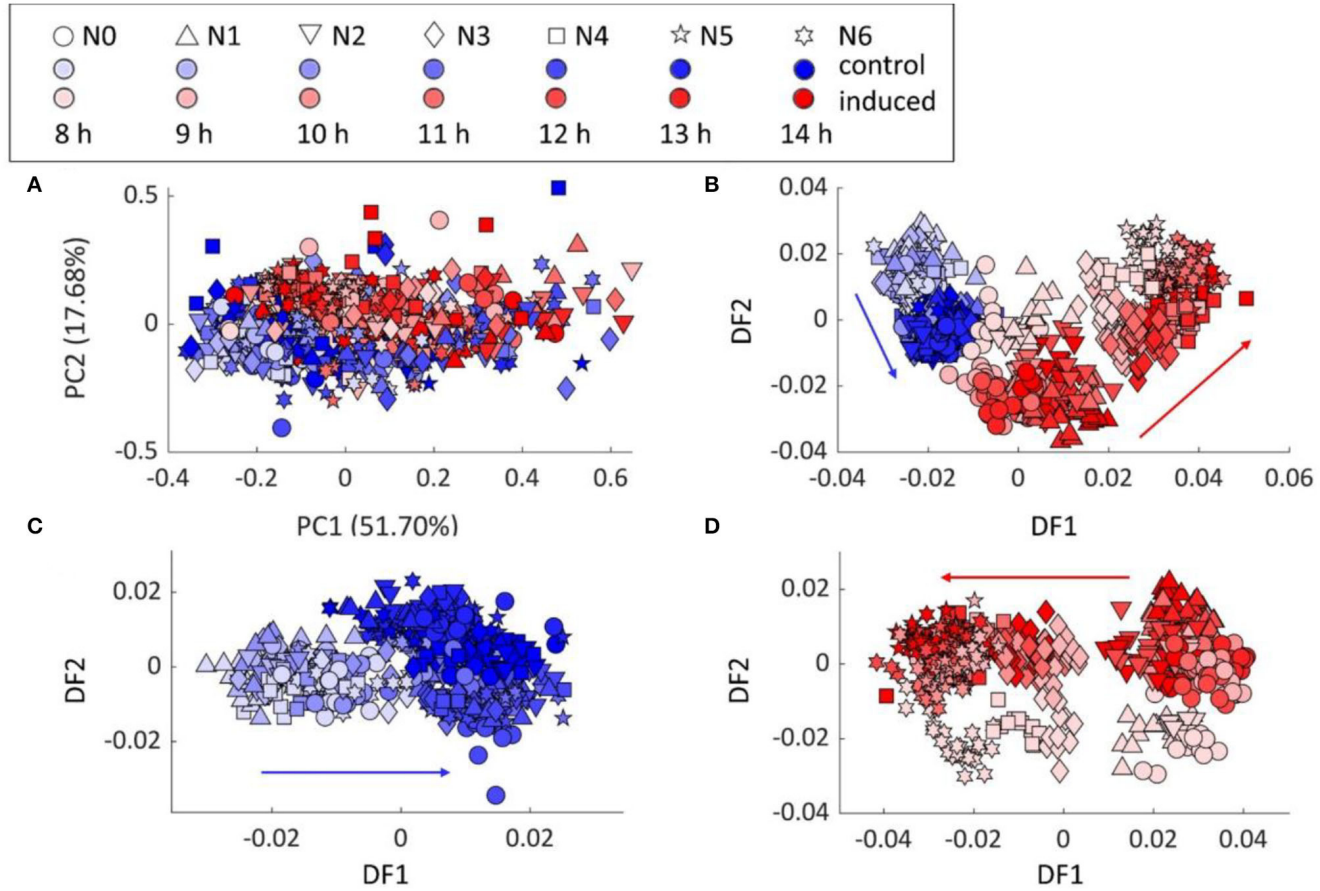
Chemometric analysis of the FT-IR spectral data: **(A)** PCA scores plot of all samples, **(B)** PC–DFA scores plots of all samples, **(C)** control samples, and **(D)** induced samples. Red arrows show the trend detected for the induced bacterial strains from N0 to N6, while the blue arrows indicate the effect of incubation time on a control set which shows no pattern linked to the protein production or the different examined strains.

PCA scores plot of FT-IR data from various incubation time (8–14 h) still showed the separation between the control and induced samples, which agreed with the previous results (Figure 2A). The PC–DFA scores plot, using 25 principal components (PCs) accounting for 99.79% of the TEV, in Figure 2B further illustrated the discrimination of 3 main groups, i.e., control samples, induced samples with low (N0–2) and high (N3–6) *cytb*_5_ copy numbers. The control and induced sample groups were also analyzed separately by PC–DFA, using 25 PCs accounting for 99.82 and 99.79% of TEV, respectively. The PC–DFA scores plot of the control set (Figure 2C), only displayed the separation of the samples according to the incubation time, which is because of the biochemical changes that are generally linked to the cells going through the various growth phases. Unlike the control samples, the PC–DFA scores plot (Figure 2D) of the induced samples displayed a gradient of discrimination among all 7 strains from N0 to N6 according to DF1 axis. Moreover, the 8-h incubation set also discriminated from the other incubation times according to DF2 axis, indicating the similarity within the groups of 9–14 h induction. Therefore, 9 h was selected as the optimum-inducing time for all the following experiments to study the metabolic changes resulting from the protein production, as it requires less incubation time and will also be more cost-effective for potentially future large-scale studies.

### Detection and Identification of CYT b_5_

**Figure 3:**
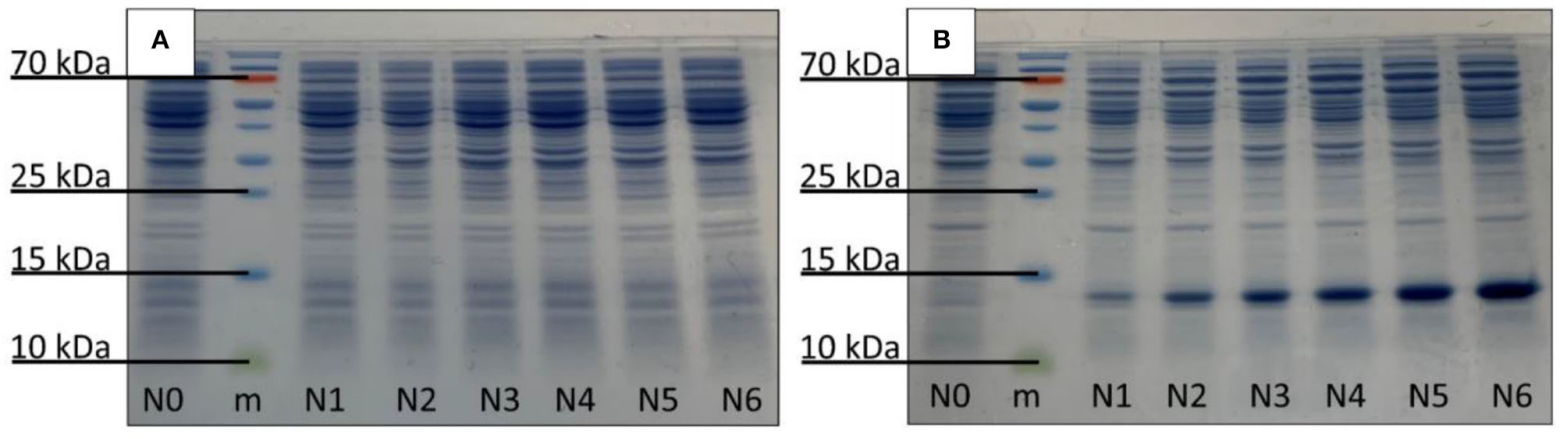
Detection of Cyt b_5_ production *via* SDS–PAGE analysis: **(A)** control and **(B)** thermally induced *E. coli* N4830-1 strains, N0–N6 carrying 0–6 copies of Cyt b_5_ gene, respectively. The protein marker is labeled as m.

The production of Cyt b_5_ was confirmed in the relevant strains using SDS–PAGE technique. The results showed that there were strong visible bands (13 kDa) in the thermally induced cultures, as expected (Figure 3B), while these were not detected in the control samples (Figure 3A). The detected protein bands were further identified by the western blot analysis (Supplementary Figure S4), which illustrated the western blot result of bacterial samples (N0–N6) with a protein ladder (m) showing bands of Cyt b_5_ at the expected position in all 6 producing strains (N1–N6). A non- Cyt b_5_ producing strain such as N0 showed no band in that region.

### Relative Quantification of Cyt b_5_

#### SDS–PAGE Method

**Figure 4:**
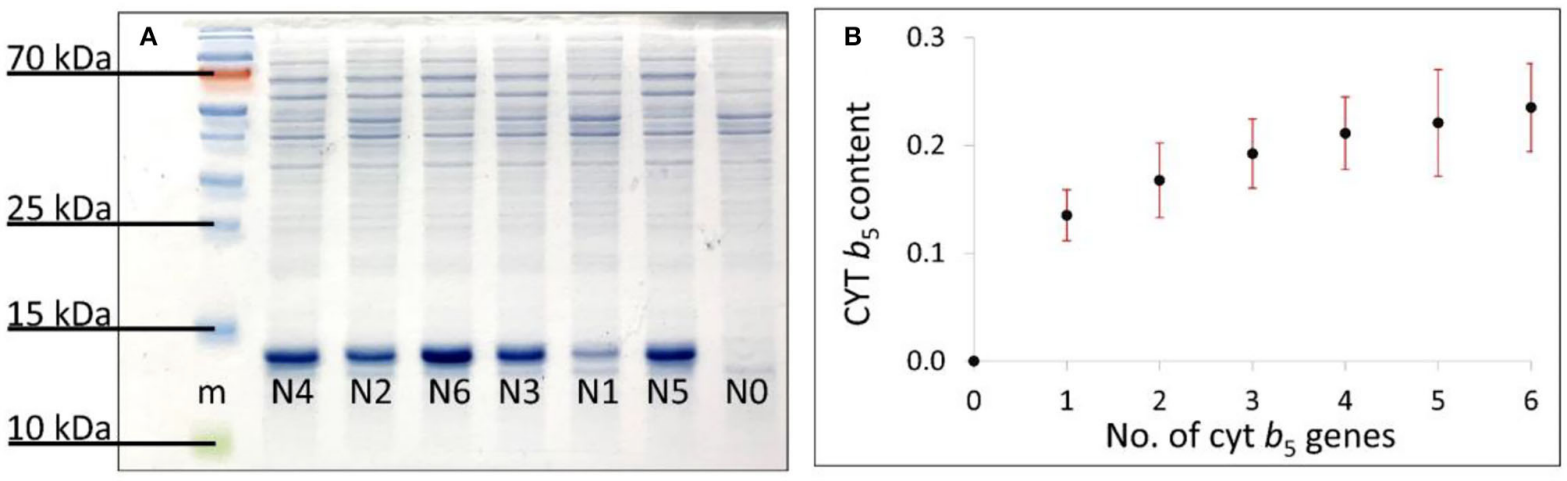
**(A)** SDS–PAGE gel of induced bacterial strains N0–N6 in a random order and **(B)** the plot of an average Cyt b_5_ bands' density from 7 biological replicates against numbers of *cytb*_5_ genes. SDs are shown as red bars.

The total protein bands from the SDS–PAGE gel were used for normalization purposes. The amount of Cyt b_5_ detected was presented as the average of the 7 biological replicates and plotted against its copy numbers (0–6) (Figure 4) showing an increasing amount of Cyt b_5_ being produced and directly linked with the gene copy number from 0 to 6 copies, as expected.

#### Colorimetric Method

**Figure 5:**
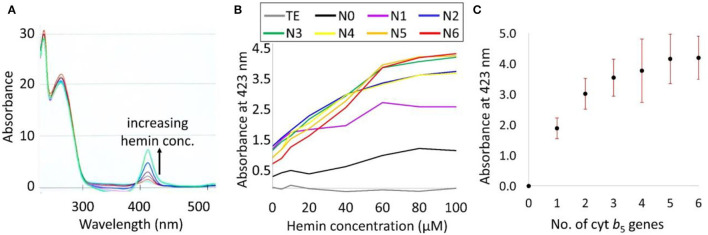
**(A)** UV-vis spectra of bacterial lysates of a strain N6 with 0–100 μM hemin supplementation showing an increase in the Soret absorption peak as indicated by a black arrow. **(B)** A plot of the Soret absorption of TE buffer (blank) and bacterial strains N0–N6 with hemin supplementation. **(C)** A plot of an average absorbance at a Soret peak representing Cyt b_5_ content from 7 biological replicates against numbers of *cytb*_5_ genes. SDs are shown as red bars.

In order to improve the accuracy of the Cyt b_5_ quantification based on its absorption at the Soret peak (423 nm), various concentrations of hemin (0–100 μM) were supplemented to determine the optimal level required. The results in Figure 5A show a higher level of the Soret absorption in the N6 strain with external hemin supplementation. Moreover, the plot in Figure 5B identifies the saturation point of hemin to be at 60 μM as any further increase in hemin concentration did not change the absorption value at 423 nm. Using the established optimum concentration of hemin (60 μM), an increasing trend of Cyt b_5_ content was detected and correlated directly with increasing gene copy numbers, as shown in Figure 5C.

## Discussion

### Bacterial Culture

All the bacterial strains illustrated comparable growth profiles at 37°C incubation temperature (Figure 1) and did not show any adverse effects from carrying various copies of the *cytb*_5_ gene. It was noteworthy that the different number of *cytb*_5_ genes seems not to affect their overall growth behavior, and the same was also observed under Cyt b_5_ producing condition (Supplementary Table S1). However, the harvested cells illustrated different shades of color where strains N3–N6 showed a visible pale pink color while the other strains did not (Supplementary Figure S2). This is perhaps not only due to the level of Cyt b_5_, as was also reported in a previous study (Kaderbhai et al., 1992), but also to do with the limited hemin availability and its incorporation into the protein structure. Therefore, the shades of color from the pellets were not directly indicative of the level of Cyt b_5_ produced. Subsequently, a confirmation by alternative methods for relative protein quantification was required. Hence, densitometric data from SDS–PAGE and colorimetric method using the Soret peak was employed, which are discussed in the following sections.

### Reproducibility and the Chemometrics Analysis

To test the reproducibility of the process, these bacteria were cultured three times in different batches and on three different days. For all batches, the PCA scores plots showed two main clusters according to the conditions, control, and induced groups (Supplementary Figure S5). The PC–DFA scores plots also displayed a clear trend for the induced samples according to the gene copy number from N0–N6, which was confirmed and illustrated in the findings of all the three separate batch cultures, highlighting the reproducibility of the whole process (Supplementary Figure S6). These multivariate analyses also indicated a clear change in the metabolic fingerprints of the control *vs*. induced cells.

To interpret the chemometrics data further, the loadings plots (Supplementary Figure S7) were investigated. The PC–DFA loadings plot of all samples (Supplementary Figure S7A) including the control and induced sets showed important vibrations that discriminated the samples, these included vibrations in the amide I and II regions (i.e., 1,569 and 1,626 cm^−1^), lipids (1,744 cm^−1^), and nucleic acids (1,082 cm^−1^), which highlighted significant metabolic changes due to the protein production process. While the PC–DFA scores plot of control samples (Figure 2C) displayed a separation based on the incubation time (8–14 h) according to DF1 axis, its loadings plot (Supplementary Figure S7B) highlighted the vibration in amide region, i.e., 1,628 cm^−1^ and lipids vibration at 1,749 cm^−1^ (C=O stretching) to be significantly changing. The latter vibration was only detected as significant in the control set (Supplementary Figure S7B) compared with that of the induced group (Supplementary Figure S7C), suggesting these differences to be mainly as a result of the biochemical changes in the cells because of the various incubation lengths and growth phases on bacterial membrane. In addition, the λ*P*_L_ promoter that is used for induction of this recombinant protein uses a temperature shift from 37 to 38.5°C and it is known that temperature shifts alter membrane fluidity in bacterial cell walls (Mejía et al., 1995) and this is likely to be the cause as to why there are changes in these lipid vibrations. This, we shall explore further when we analyze these bacteria using lipidomics. By contrast, the PC–DFA loadings plot of induced samples (Supplementary Figure S7C) demonstrated that the most significant changes in DF1 axis is accounted for by the variation in amide vibrations relating to the protein secondary structures, such as the vibrational bands at 1,626 and 1,569 cm^−1^ (β-sheet) and 1,658 cm^−1^ (α-helix) (Holloway and Mantsch, 1989; Kong and Yu, 2007; Brian et al., 2014). These findings suggested that the 7 bacterial strains examined in this study are mainly discriminated based on their Cyt b_5_ content, which is directly correlated to the their *cytb*_5_ gene copy number. To test these findings further, the peak height ratios of the significant amide vibrations, 1,631 cm^−1^ (β-sheet) and 1,658 cm^−1^ (α-helix) were calculated and found to be showing an increasing trend from bacterial strains N0–N6, as expected (Supplementary Figure S8). Since the majority structures of the Cyt b_5_ are β-sheets (Holloway and Mantsch, 1989), this result also indicated the different level of Cyt b_5_ production among the 7 strains.

### Relative Protein Quantification

**Figure 6:**
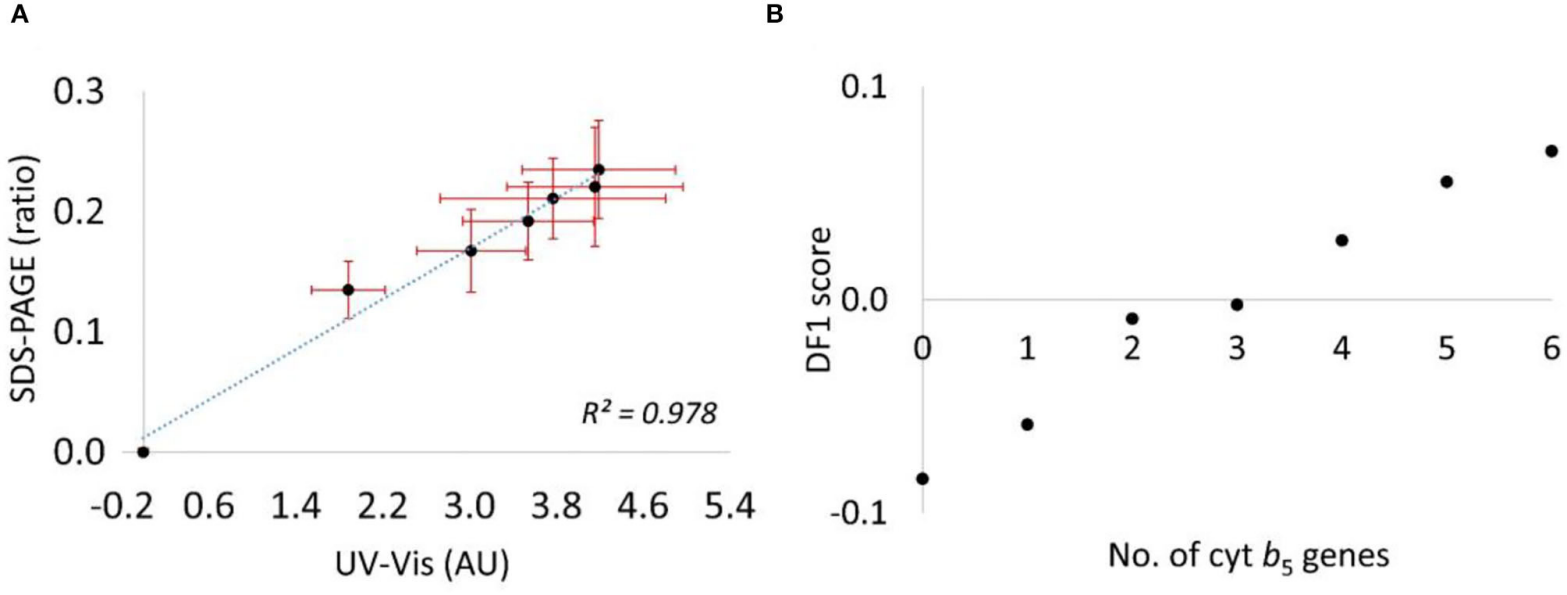
**(A)** A combination plot of *cytb*_5_ content relatively quantified by the 2 techniques, the ratio of densitometric data from an SDS–PAGE (no unit) and colorimetric data from UV-vis absorption of a Soret peak (absorbance unit, AU). **(B)** A plot of DF1 score from the PC–DFA scores plot against numbers of *cytb*_5_ gene.

The first approach was to quantify the Cyt b_5_ *via* western blot analysis, using the β-actin band for normalization. However, the Cyt b_5_ was expressed at very high levels, and resulted in saturation of the bands. Several attempts were made to correct this issue, including dilution of the protein extracts and reducing the volume of samples loaded into the gel; however, this also reduced the control bands and compromised the reproducibility of this approach (Supplementary Figure S4). Therefore, to quantify the Cyt b_5_ more reliably, the total protein bands in SDS–PAGE was used along with the random ordering of samples to avoid any bias resulting from samples' positions on the gel. With 7 biological replicates, this technique provided an increasing trend of Cyt b_5_ content similarly to that from the colorimetric method. As expected, the absorbance at Soret band increased with the concentration of supplemented hemin (0–100 μM) in Cyt b_5_-producing strains before reaching the saturation level. This indicated the lack of heme incorporation into the produced protein providing the Soret absorption, especially, in strains with higher copy numbers of the gene. The findings of densitometric analysis from SDS–PAGE and of the Soret absorption using spectroscopy for the relative Cyt b_5_ quantification are consistent as suggested in a combination plot (Figure 6A) with *R*^2^ value of 0.978. Furthermore, a plot of DF1 scores (Figure 6B) from FT-IR fingerprinting data of induced samples also provided a consistent result to those from the wet-lab protein quantitative techniques.

## Conclusion

Detection, quantification, and optimization of recombinant production is important that normally requires laborious and time-consuming techniques such as a traditional SDS–PAGE together with a western blot. The samples are usually prepared by lysing the host cells to further investigate the protein of interest. The application of FT-IR spectroscopy as a holistic metabolic fingerprinting approach has proven to be a useful and reliable approach for studying such metabolic changes in bacterial cells. The sample could be readily studied by centrifugation of the bacterial culture without the need for a cell lysis step. In this study, by combining FT-IR with chemometric methods, we could separate the control from the temperature-induced samples. Furthermore, the PC–DFA scores plots of induced samples allowed for clear separation of the strains, from N0 to N6, based on their Cyt b_5_ protein content. Such trend was not observed in the control set; nevertheless, the metabolic effects from temperature and incubation time on bacterial membrane was apparent by detected changes in other biomolecules such as the lipids vibration at 1,749 cm^−1^. Besides, an increase in Cyt b_5_ content along with its copy numbers was confirmed by two traditional methods, the use of densitometric data from SDS–PAGE together with the total protein normalization and the colorimetric method by measuring the Soret absorption. The latter approach required a supplementation of hemin into the lysates for the actual representation of the hemoprotein by its Soret absorption. However, to achieve a clearer understanding of the metabolic burden of recombinant Cyt b_5_ production in the examined strains, such as the effect on the lipid profile in the case of exported recombinant protein through bacterial membrane, future studies should perhaps focus on using MS-based metabolomics approaches such as metabolic profiling (extracted intracellular metabolites), footprinting (extracellular metabolites), and lipidomics (of membrane lipids) to investigate further the possible bottleneck(s) of the recombinant protein production. The findings of such approaches may provide a clearer picture of what goes on inside the host cell and may potentially support the design and optimization of the media, and guide future genetic engineering strategies leading to a more efficient production system.

## Data Availability Statement

The raw data supporting the conclusions of this article will be made available by the authors, without undue reservation.

## Author Contributions

TT: experimental design, sample collection and preparation, protein quantification, FT-IR data analysis, and data interpretation. NK and JG: genetic engineering of the strains and growth condition optimisation. RG: experimental design, data interpretation, and manuscript preparation. HM: principal investigator, experimental design, data interpretation, and manuscript preparation. All authors read and approved the final manuscript.

## Conflict of Interest

The authors declare that the research was conducted in the absence of any commercial or financial relationships that could be construed as a potential conflict of interest.

## Publisher's Note

All claims expressed in this article are solely those of the authors and do not necessarily represent those of their affiliated organizations, or those of the publisher, the editors and the reviewers. Any product that may be evaluated in this article, or claim that may be made by its manufacturer, is not guaranteed or endorsed by the publisher.
