# Supplementary material for: Metabolic Fingerprint Analysis of Cytochrome b5-producing E. coli N4830-1 Using FT-IR Spectroscopy
Source: Front Microbiol. 2022 Jun 22;13:874247. doi: 10.3389/fmicb.2022.874247 (PMC9257212; doi:10.3389/fmicb.2022.874247)
Supplement: Supplementary file 1 [file Data_Sheet_1.docx]

Supplementary Material

**Supplemental Figure 1.** Biosynthesis pathways of iridoid glycoside in *R. glutinosa*.

**Supplemental Figure 2.** Methylation banding pattern in *R. glutinosa*. a: The presence of band in H and M was considered to be no-methylation (class I); b: The presence of band only in H was considered to be DNA hemi-methylation (class II); c: The presence of band only in M was considered to be DNA full methylation (class III).

**Supplemental Figure 3.** cDNA and amino acid sequences of target genes in *R. glutinosa*. **(A)**, **(B)**, **(C)**, **(D)** and **(E)** represent cDNA and amino acid sequences of *DXS* (MG764508), *DXR* (MG764509), *GPPS* (MG770219), *G10H* (MK559439) and *10HGO* (MH102394) in *R. glutinosa*, respectively.

**Supplemental Figure 4.** The genetic structure of iridoid glycoside synthetase genes in *R. glutinosa*.

**Supplemental Figure 5.** The DNA sequence of *DXS* in *R. glutinosa*. Intron sequences are shown with the underlined regions.

**Supplemental Figure 6.** Phylogenetic analysis of iridoid glycoside synthetases in plants. **(A)**, **(B)**, **(C)**, **(D)** and **(E)** respectively represent the phylogenetic tree of h[omologous](javascript:;) proteins for DXS, DXR, GPPS, G10H and 10HGO of *R. glutinosa*.

**Supplemental Figure 7.** Sequence alignment of iridoid glycoside synthetases in plants. **(A)**, **(B)**, **(C)**, **(D)** and **(E)** respectively represent the alignment among h[omologs](javascript:;) for DXS, DXR, GPPS, G10H and 10HGO of *R. glutinosa*. Red underline indicates conservative domain.

**Supplemental Figure 1**

**
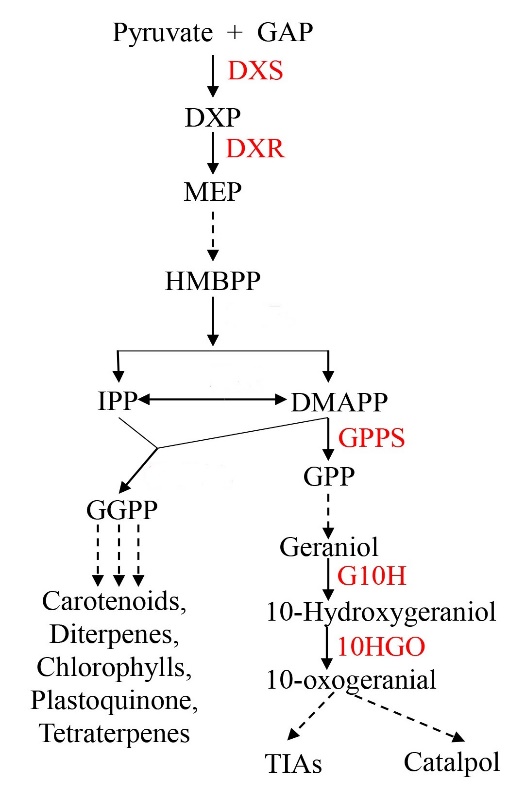
**

**Supplementary Figure 1.** Biosynthesis pathways of iridoid glycoside in *R. glutinosa*. Red indicates enzymes involved in this study.

**Supplemental Figure 2**


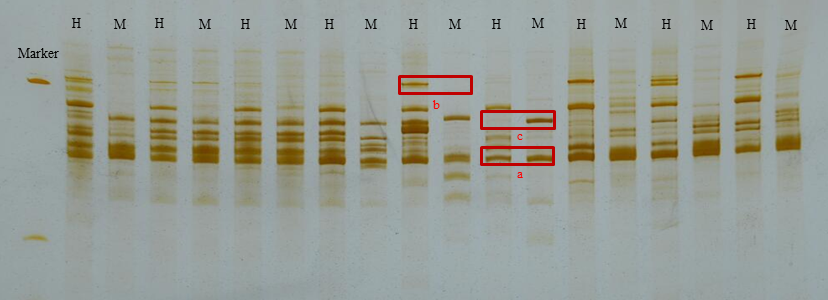


**Supplemental Figure 2.** Methylation banding pattern in *R. glutinosa*. a: The presence of band in H and M was considered to be no-methylation (class I); b: The presence of band only in H was considered to be DNA hemi-methylation (class II); c: The presence of band only in M was considered to be DNA full methylation (class III).

**Supplemental Figure 3**


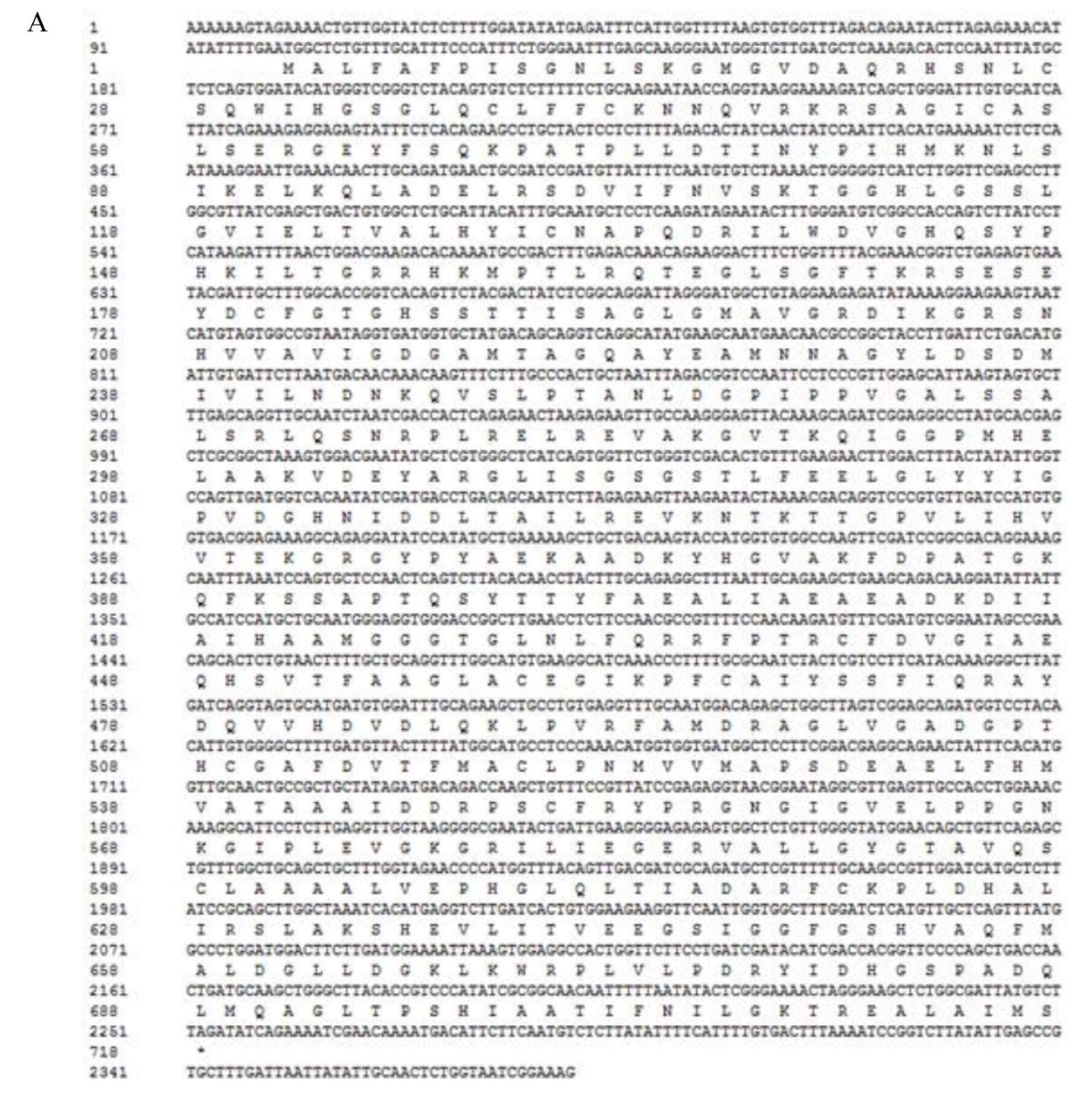


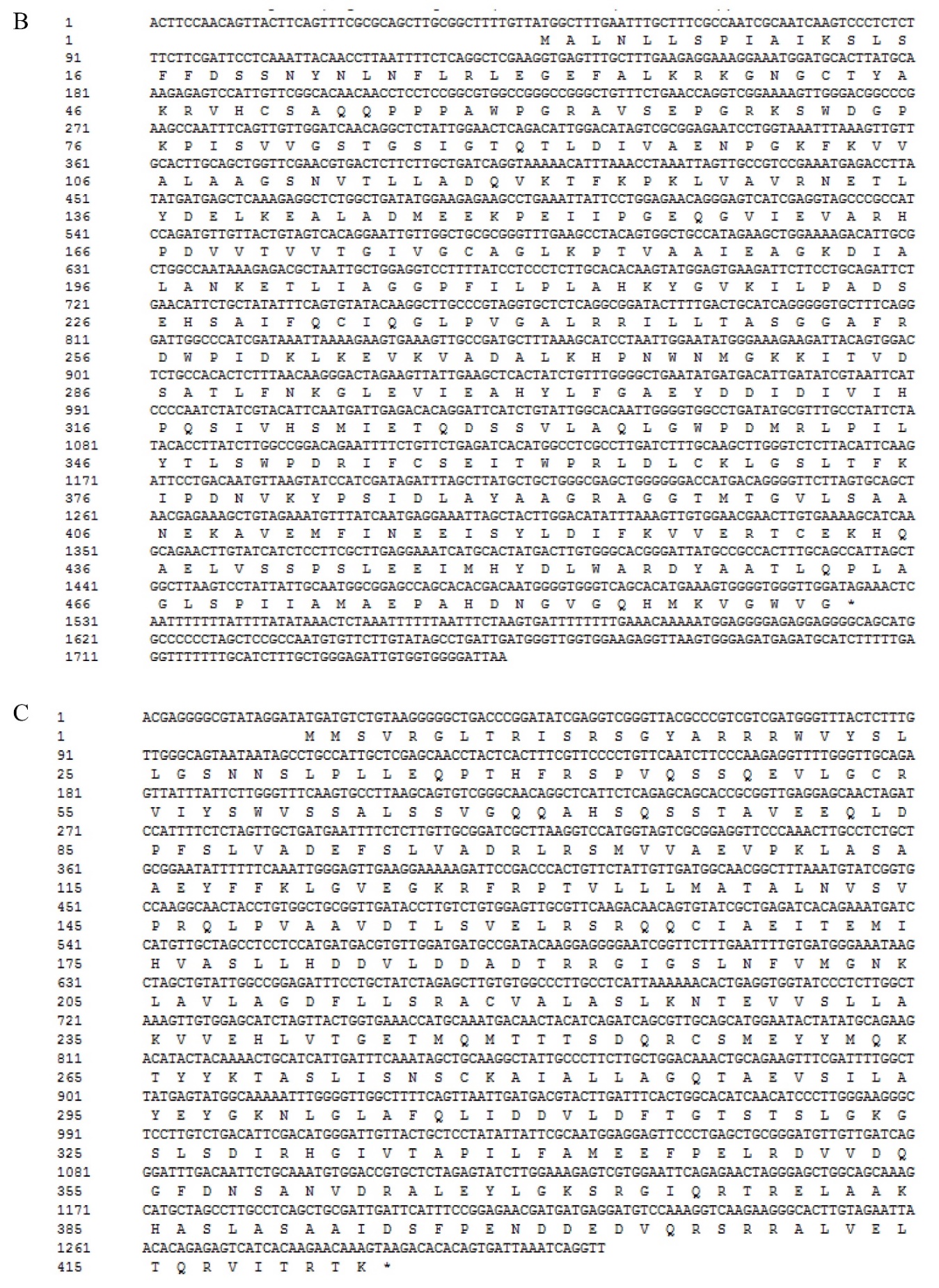


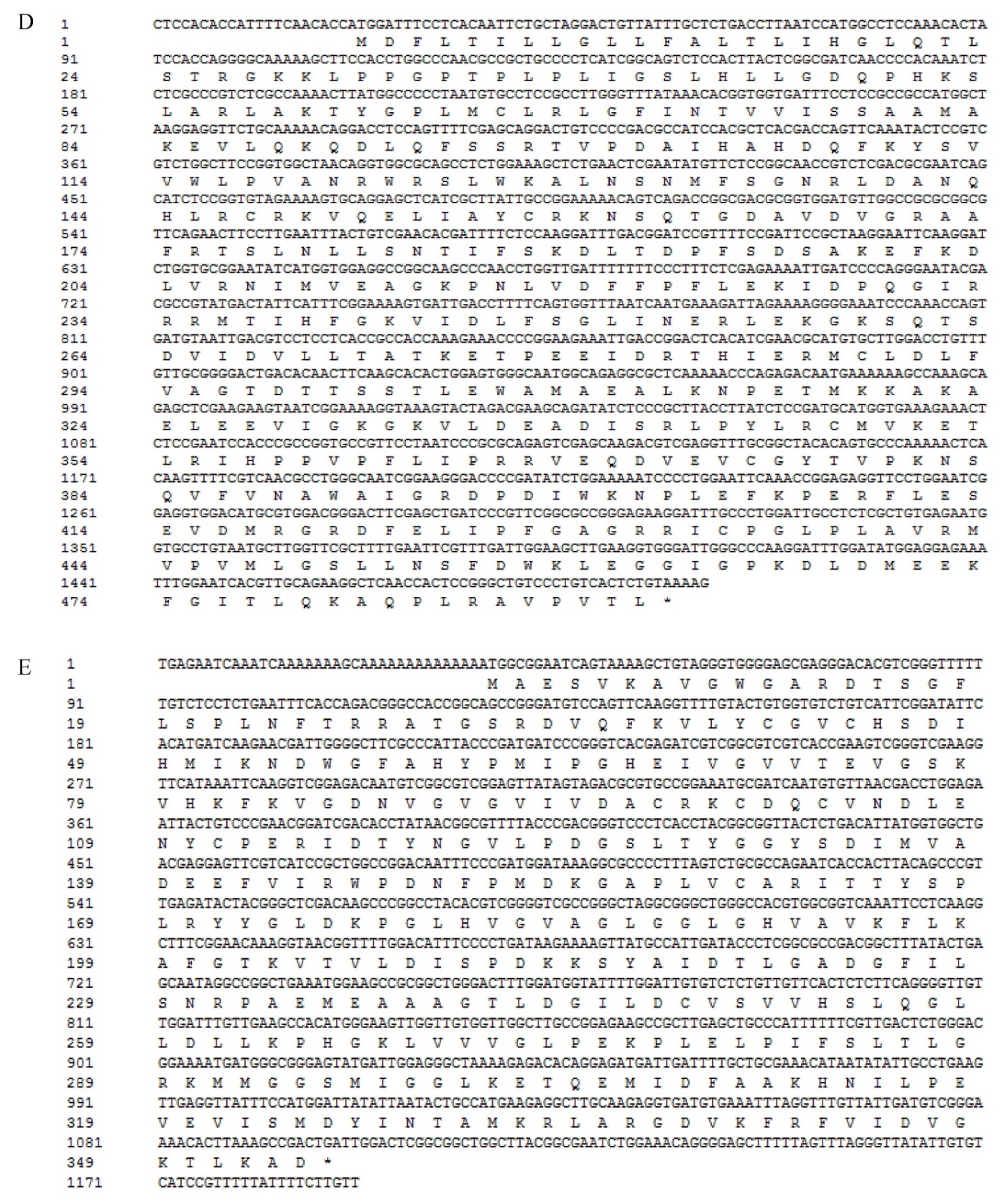


**Supplemental Figure 3.** cDNA and amino acid sequences of target genes in *R. glutinosa*. **(A)**, **(B)**, **(C)**, **(D)** and **(E)** represent cDNA and amino acid sequences of *DXS* (MG764508), *DXR* (MG764509), *GPPS* (MG770219), *G10H* (MK559439) and *10HGO* (MH102394) in *R. glutinosa*, respectively.

**Supplemental Figure 4**


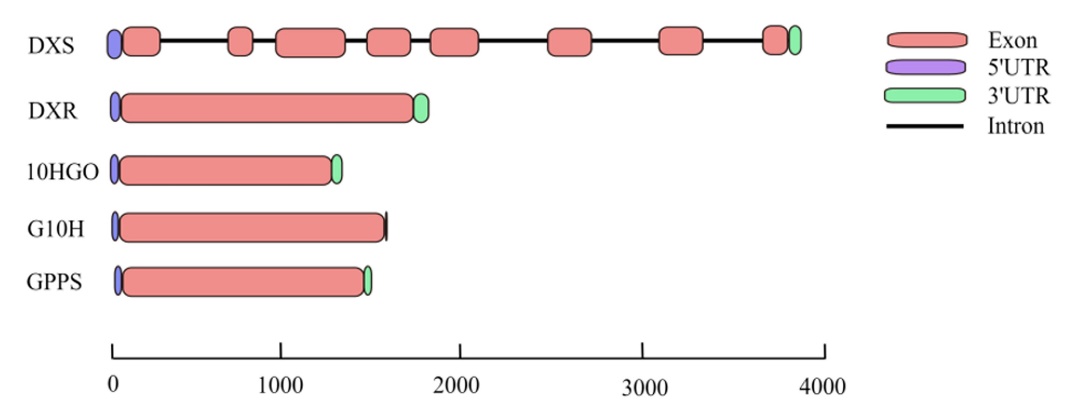


**Supplemental Figure 4.** The genetic structure of iridoid glycoside synthetase genes in *R. glutinosa*.

**Supplemental Figure 5**

**
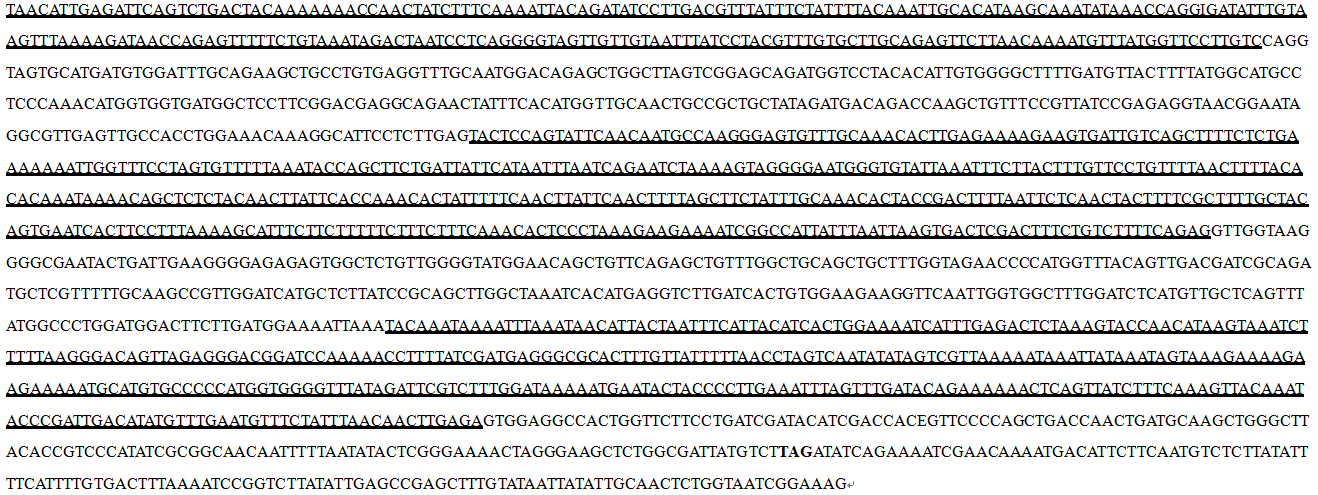

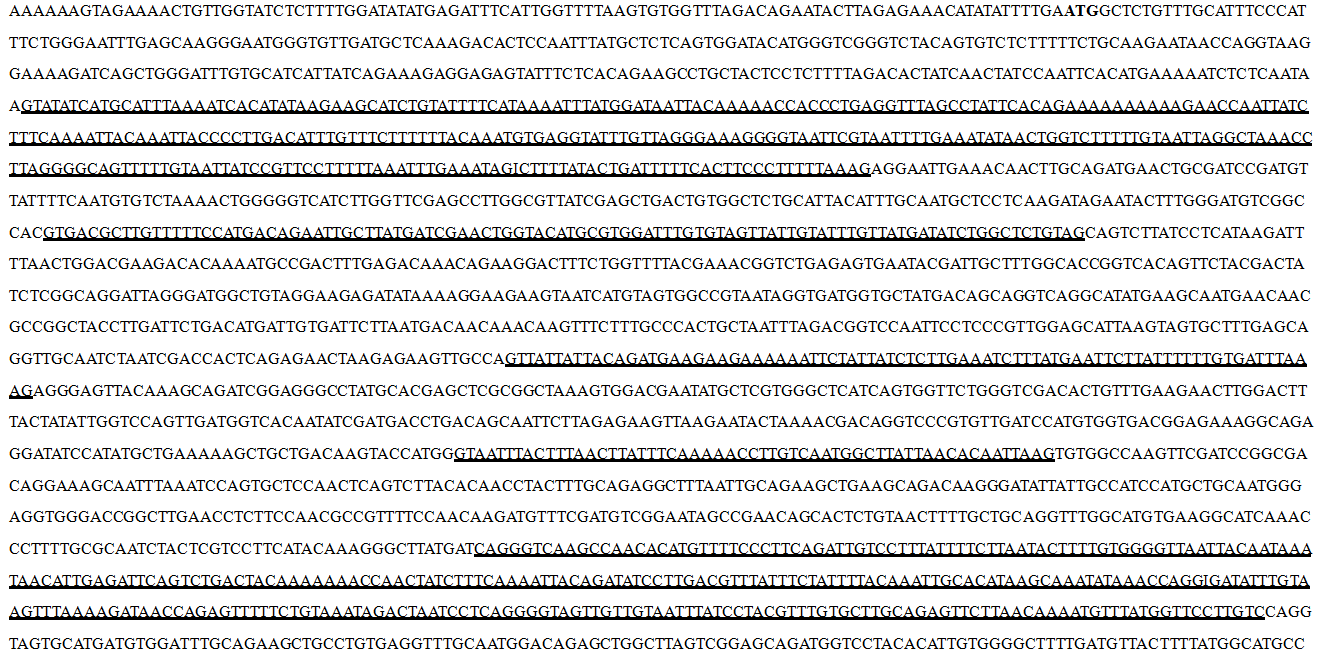
 Supplemental Figure 5.** The DNA sequence of *DXS* in *R. glutinosa*. Intron sequences are shown with the underlined regions.

**Supplemental Figure 6**


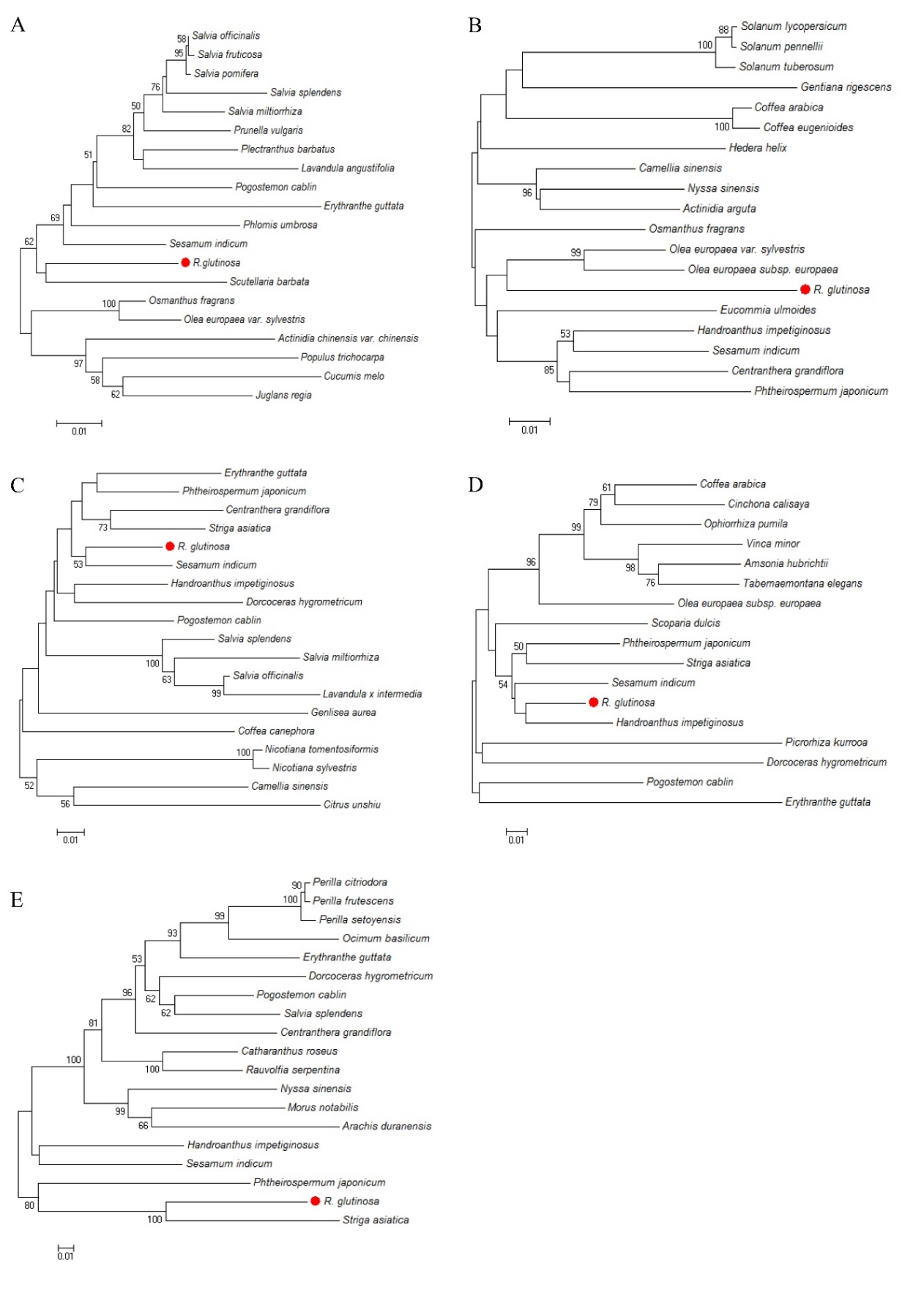


**Supplemental Figure 6.** Phylogenetic analysis of iridoid glycoside synthetases in plants. (A), (B), (C), (D) and (E) respectively represent the phylogenetic tree of homologous proteins for DXS, DXR, GPPS, G10H and 10HGO of *R. glutinosa*.

**Supplemental Figure 7**

**
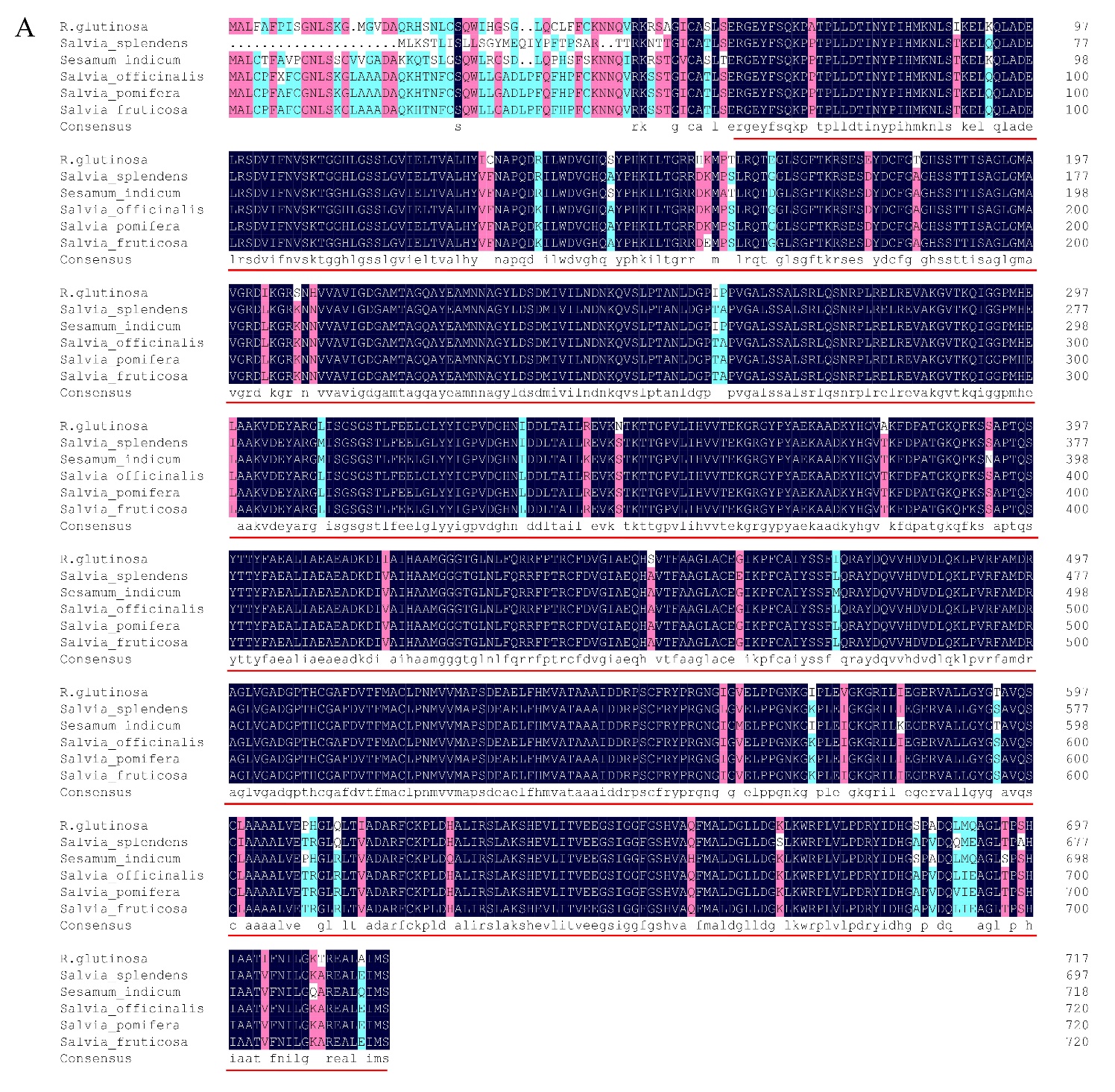
**

**
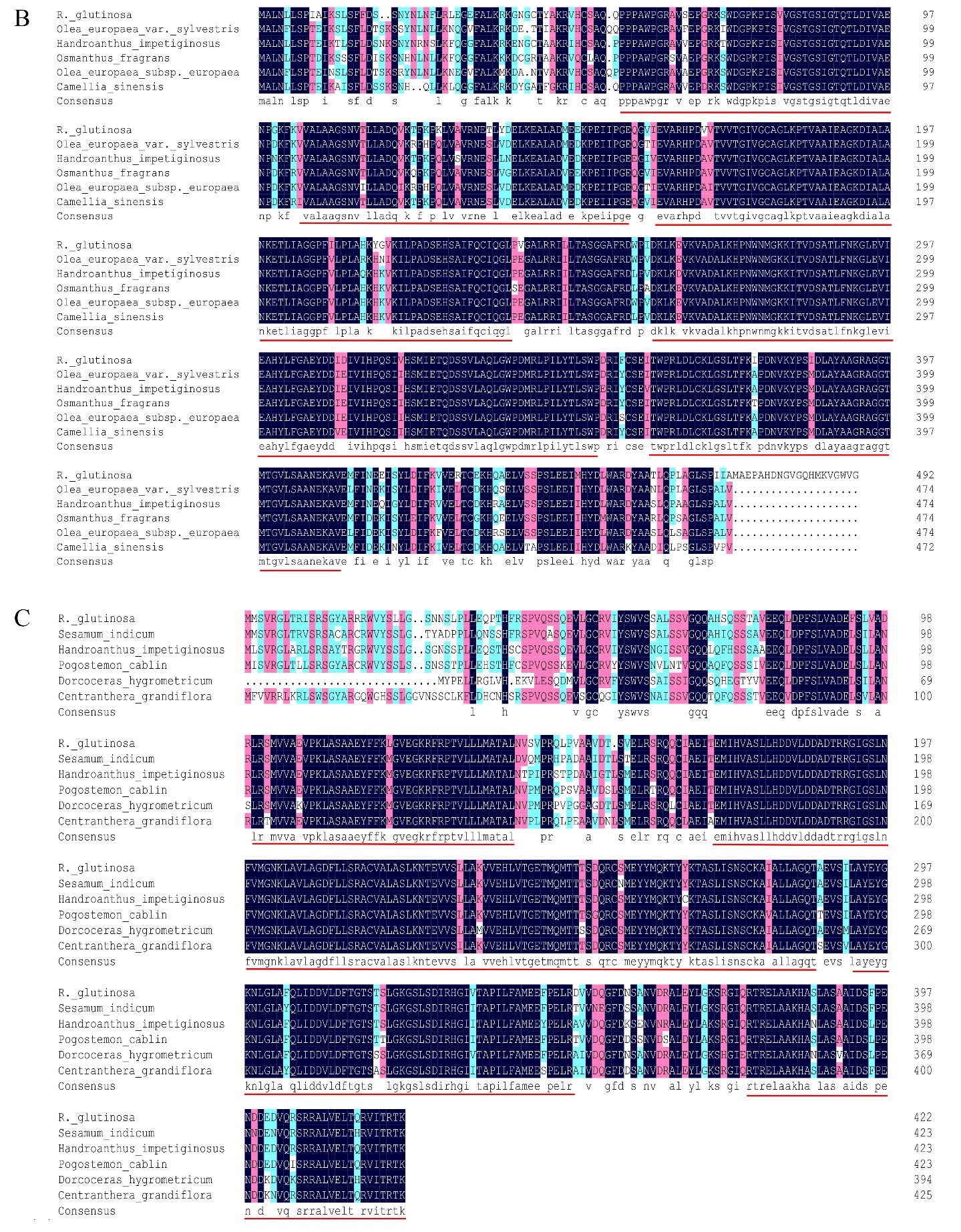
**

**
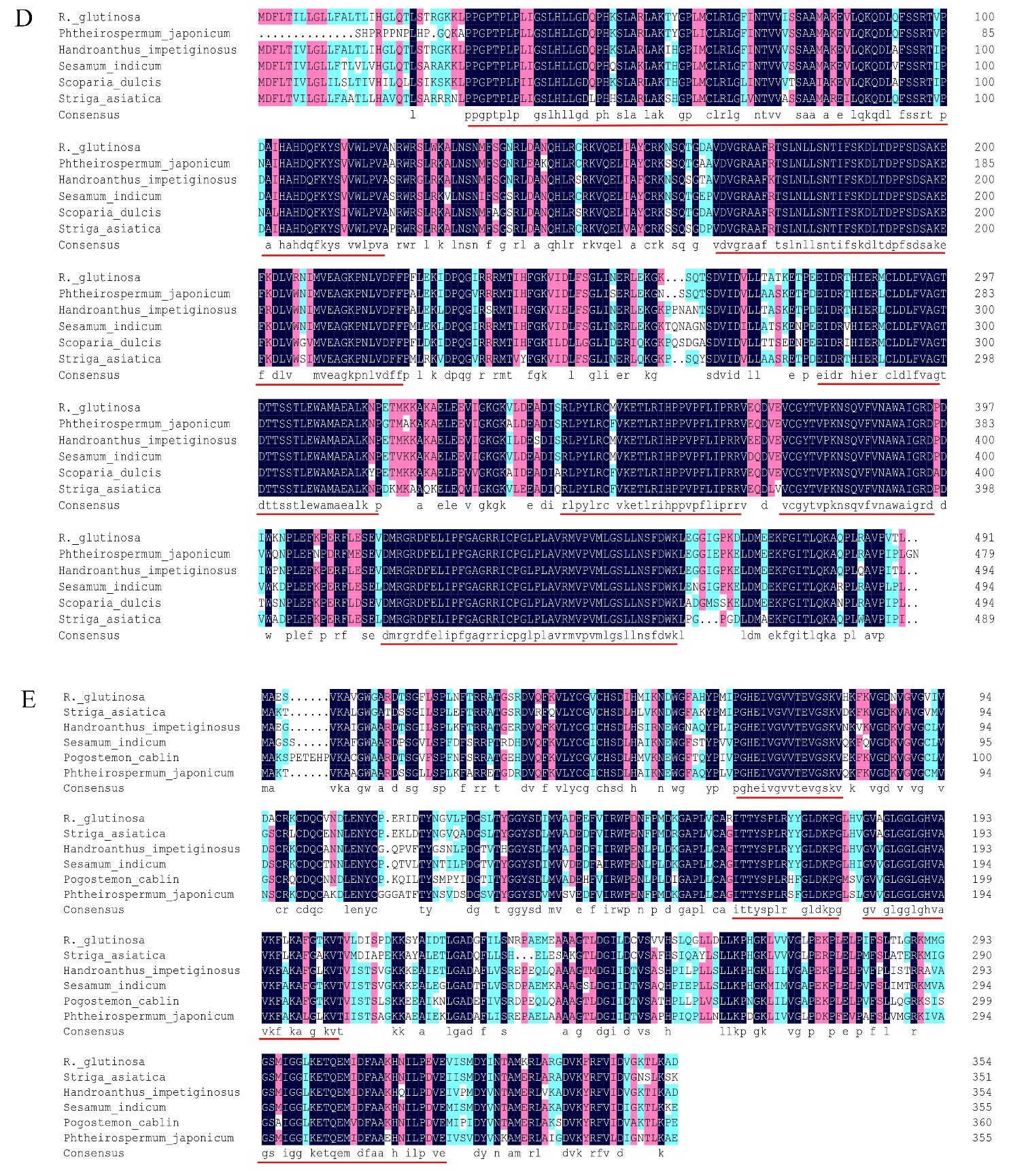
**

**Supplemental Figure 7.** Sequence alignment of iridoid glycoside synthetases in plants. **(A)**, **(B)**, **(C)**, **(D)** and **(E)** respectively represent the alignment among h[omologs](javascript:;) for DXS, DXR, GPPS, G10H and 10HGO of *R. glutinosa*. Red underline indicates conservative domain.
